# Circulating Novel Adipokines in Critically Ill Patients with and Without Sepsis

**DOI:** 10.3390/biomedicines14061324

**Published:** 2026-06-11

**Authors:** Vassiliki Giannopoulou, Ioannis Ilias, Chrysi Keskinidou, Charikleia S. Vrettou, Olga Kampouropoulou, Nikolaos S. Lotsios, Matina Kardara, Kostas A. Papavassiliou, Georgios-Ioannis Poupouzas, Vasileios Issaris, Anastasia Kotanidou, Alice G. Vassiliou, Ioanna Dimopoulou

**Affiliations:** 1 1st Department of Critical Care Medicine, Evangelismos Hospital, School of Medicine, National and Kapodistrian University of Athens, 10676 Athens, Greece; vaso.giannop88@gmail.com (V.G.); chrysakes29@gmail.com (C.K.); kliovrettou@med.uoa.gr (C.S.V.); olgakampou@yahoo.com (O.K.); nlotsios@med.uoa.gr (N.S.L.); kardara.matina@gmail.com (M.K.); poupouzas.gewr.kw@gmail.com (G.-I.P.); vasilisiss@gmail.com (V.I.); akotanid@med.uoa.gr (A.K.); alvass@med.uoa.gr (A.G.V.); idimop@med.uoa.gr (I.D.); 2 Department of Endocrinology, Hippokration Hospital, 11527 Athens, Greece; 3 First University Department of Respiratory Medicine, ‘Sotiria’ Chest Hospital, School of Medicine, National and Kapodistrian University of Athens, 11527 Athens, Greece; kpapavassiliou@gmail.com; 4 Department of Biological Chemistry, School of Medicine, National and Kapodistrian University of Athens, 11527 Athens, Greece

**Keywords:** adipokines, omentin-1, vaspin, chemerin, sepsis, ICU, critically ill, mortality, biomarkers

## Abstract

**Background/Objectives**: Adipokines are candidate biomarkers in critical illness due to their roles in immunity and metabolism, both profoundly altered in sepsis. Omentin-1, vaspin, and chemerin have been studied in selected septic cohorts, but not concurrently in a heterogeneous ICU population including both septic and non-septic patients. **Methods**: Prospective observational cohort of 200 consecutive ICU patients with 28-day follow-up. Biomarkers were measured by ELISA within 24 h of admission. Analyses included Mann–Whitney U tests, Spearman correlations, ROC curves, and logistic regression with APACHE II and SOFA as comparators. **Results**: Vaspin was significantly higher in septic versus non-septic patients (406.4 [190.0–799.6] vs. 275.8 [101.8–559.8] pg/mL; *p* = 0.009). Omentin-1 was elevated in 28-day non-survivors (34.4 [22.5–56.1] vs. 25.1 [15.0–48.4] ng/mL; *p* = 0.037; AUROC 0.599), but lost significance after APACHE II adjustment (*p* = 0.295). Chemerin trended lower in non-survivors (*p* = 0.099); in septic patients, it correlated inversely with SOFA (r = −0.43) and lactate (r = −0.40), both *p* < 0.001. IL-6 and IL-10 were higher in non-survivors; IL-10 predicted 28-day mortality (AUROC 0.783), comparable to APACHE II (0.785). **Conclusions**: Vaspin distinguishes sepsis in mixed ICU populations. Omentin-1 shows a severity-driven association with mortality that does not survive APACHE II adjustment (AUROC 0.599, poor standalone discrimination), while chemerin inversely tracks hypoperfusion markers in septic patients, suggesting a potential counter-regulatory role requiring mechanistic confirmation. Individually, these adipokines do not add prognostic value beyond established severity scores, but their biological orthogonality to classical cytokines warrants exploration in multi-marker panel studies.

## 1. Introduction

Sepsis, defined as life-threatening organ dysfunction caused by a dysregulated host response to infection [1], remains the foremost cause of in-hospital death in intensive care units (ICUs), accounting for an estimated 11 million deaths annually worldwide [2]. Reliable biomarkers for early diagnosis, severity stratification, and prognostication in unselected critically ill populations remain an unmet clinical need despite over 250 candidates having been investigated [3].

Classical inflammatory markers—C-reactive protein, procalcitonin, interleukin (IL)-6, and IL-10—are routinely available and their associations with sepsis severity and mortality are firmly established. IL-6 correlates with shock progression and adverse outcomes across multiple cohorts [4], and IL-10 similarly predicts mortality and informs therapeutic decisions [5]. These associations, however, are well-known and not the focus of the present work. Both cytokines are non-specific, being elevated in any major inflammatory state, which limits their discriminatory value in heterogeneous ICU populations [6].

Adipokines—a rapidly expanding family of bioactive proteins secreted primarily by visceral adipose tissue (VAT)—offer a potentially complementary approach. VAT functions as an active endocrine and immune organ whose secretory output is profoundly altered during critical illness through mechanisms distinct from the cytokine storm, including peroxisome proliferator-activated receptor-γ downregulation, protease-mediated processing, and receptor expression changes [7,8]. Three adipokines have been the focus of prior investigation in septic cohorts but have not been evaluated simultaneously in a broadly inclusive ICU population:

Omentin-1 (intelectin-1), an anti-inflammatory adipokine inhibiting nuclear factor kappa β (NF-κB) and lipopolysaccharide (LPS)-induced macrophage activation, has been shown to be elevated in septic patients, with higher concentrations associating with worse outcomes [9,10]. The prevailing hypothesis is a compensatory upregulation proportional to inflammatory burden rather than a directly harmful effect.

Vaspin (visceral adipose tissue-derived serpin), a serine protease inhibitor with insulin-sensitizing and anti-inflammatory properties, has been reported to be approximately threefold higher in septic than in non-septic surgical ICU controls [11], suggesting a diagnostic rather than prognostic role.

Chemerin is a multifunctional adipokine that recruits antigen-presenting cells via the chemokine-like receptor 1 (CMKLR1), exerts direct antimicrobial activity, and modulates insulin sensitivity. Elevated circulating chemerin has been reported in peritoneal sepsis [12] and in critically ill patients with Gram-positive co-infections [13], whilst its kinetics during the first week of sepsis may carry prognostic information [14].

We report a cross-sectional analysis of 200 unselected consecutive ICU admissions in which omentin-1, vaspin, and chemerin were simultaneously measured alongside IL-6 and IL-10. Our primary aims were: (i) to determine which adipokines distinguish septic from non-septic critically ill patients at admission; (ii) to characterize their correlations with validated severity indices; and (iii) to evaluate their univariate discriminatory performance for ICU and 28-day mortality alongside established comparators.

## 2. Materials and Methods

### 2.1. Study Design and Population

This was a prospective observational cohort study of 200 consecutive adult patients (≥18 years) on mechanical ventilation, who were admitted to a mixed medical-surgical ICU at a tertiary center, with a single biomarker measurement at admission and prospective 28-day outcome follow-up. We excluded patients who were transferred from another ICU, patients on mechanical ventilation in the ward >48 h. Pregnant women were also excluded, as well as subjects with contagious diseases (HIV, hepatitis) and those with an expected length of stay in the ICU < 3 days. Patients with active autoimmune disease requiring systemic immunosuppression, solid organ transplantation, or pre-existing chronic inflammatory conditions that independently elevate adipokines (e.g., active rheumatoid arthritis, inflammatory bowel disease) were excluded. Patients with obesity, diabetes, hypertension, chronic kidney disease, or corticosteroid therapy were not excluded because these represent typical ICU comorbidities; their distribution is presented in Table 1. No patients were excluded for missing biomarker data (complete case rate 100% for all five ELISA markers). The protocol was approved by the Research Ethics Committee of “Evangelismos” General Hospital of Athens (protocol code 346-5/10/2022). All patients’ next-of-kin provided written informed consent for participation. Only the admission blood draw was analyzed. Patients were classified as septic if they met Sepsis-3 criteria [1]: confirmed or suspected infection with an acute SOFA increase of ≥2 points. The non-septic group comprised patients admitted for non-infectious critical illness (post-operative care, trauma, acute cardiorespiratory failure) or with suspected infection not meeting full Sepsis-3 criteria at the time of the admission blood draw. The study was conducted in accordance with the Declaration of Helsinki.

### 2.2. Biomarker Measurement

Venous blood was collected within 24 h of ICU admission. Serum was separated by centrifugation at 2000× *g* for 15 min and stored at −80 °C until batch analysis. Enzyme-linked immunosorbent assay (ELISA) was employed to measure patients’ serum adipokine and cytokine levels. The following kits were employed: Chemerin (R&D Systems, Inc. Bio-Techne, Minneapolis, MN, USA); detection limit, 7.8 pg/mL; intra-assay coefficient of variation (CV), 3.9%; inter-assay CV, 7.3%. Vaspin (R&D Systems, Inc. Bio-Techne, Minneapolis, MN, USA); detection limit, 14.6 pg/mL; intra-assay CV, 3.6%, inter-assay CV, 6.3%. Omentin-1 (R&D Systems, Inc. Bio-Techne, Minneapolis, MN, USA); detection limit, 0.38 ng/mL; intra-assay coefficient of variation (CV), 4.8%, inter-assay CV, 4.8%. Interleukin (IL)-6 (R&D Systems, Inc. Bio-Techne, Minneapolis, MN, USA), detection limit, 0.7 pg/mL; intra-assay coefficient of variation (CV), 2.6%, inter-assay CV, 4.5%, and IL-10 (Biolegend, Inc., San Diego, CA, USA); detection limit, 2 pg/mL; intra-assay CV, 5.7%, inter-assay CV, 7.3%. Samples with visible hemolysis (visual inspection) were excluded before analysis; no hemolysed samples were encountered. Outliers were defined as values exceeding the 99.5th percentile; no biomarker measurement required exclusion on this basis. Routine laboratory parameters (CRP, lactate, creatinine, hemoglobin, procalcitonin) were measured contemporaneously by certified clinical laboratories. There were no missing values for any of the five ELISA markers (complete case rate 100%).

### 2.3. Clinical Variables and Outcomes

APACHE II and SOFA scores were computed from worst values within the first 24 h. The primary outcome was 28-day all-cause mortality; the secondary outcome was ICU mortality. All patients were followed for a minimum of 28 days.

### 2.4. Statistical Analysis

Continuous variables are reported as median [IQR]; categorical variables as n (%). Between-group comparisons used the two-sided Mann–Whitney U test (continuous) or Pearson chi-squared test (categorical). Spearman rank correlation coefficients (r) assessed monotonic associations between biomarker concentrations and severity indices. Given five primary biomarker group comparisons (three adipokines, two cytokines), a Bonferroni-corrected significance threshold of *p* < 0.01 was applied alongside the conventional *p* < 0.05. To explore whether biomarker associations with 28-day mortality persisted after adjustment for baseline severity, logistic regression was performed entering log-transformed biomarker concentrations with APACHE II or SOFA as co-predictors. Supplementary multivariable models additionally adjusted for corticosteroid use during ICU stay, diabetes status, and chronic kidney disease as covariates known to influence adipokine biology. Discriminatory ability for mortality was evaluated by the area under the ROC curve (AUROC); 95% confidence intervals were estimated by DeLong’s method. Both APACHE II and SOFA were used as internal reference standards; optimal thresholds were identified by the Youden index. There were no missing values for any of the five ELISA markers (complete case rate 100%); for covariates with sparse missing data (<4%), complete-case analyses were used. A two-tailed *p* < 0.05 was considered statistically significant for all analyses not subject to the Bonferroni correction. All analyses were performed in Python 3.11 using scipy, scikit-learn, statsmodels, pandas, and matplotlib.

## 3. Results

### 3.1. Patient Characteristics

Table 1 presents baseline data for all 200 patients, 86 (43.0%) of whom met Sepsis-3 criteria on ICU admission. Septic patients were significantly older (median 66 vs. 60 years; *p* = 0.006) and had higher severity scores (APACHE II 17 vs. 13, *p* < 0.001; SOFA 8 vs. 7, *p* < 0.001), lactate (1.7 vs. 1.1 mmol/L; *p* = 0.001), and CRP (15.4 vs. 5.8 mg/dL; *p* < 0.001). Overall ICU mortality was 31.0% (62/200); 28-day mortality was 24.5% (49/200) and was significantly higher in septic patients (33.7% vs. 17.5%; *p* = 0.014).

### 3.2. Adipokine Concentrations by Sepsis Status

**Figure 1:**
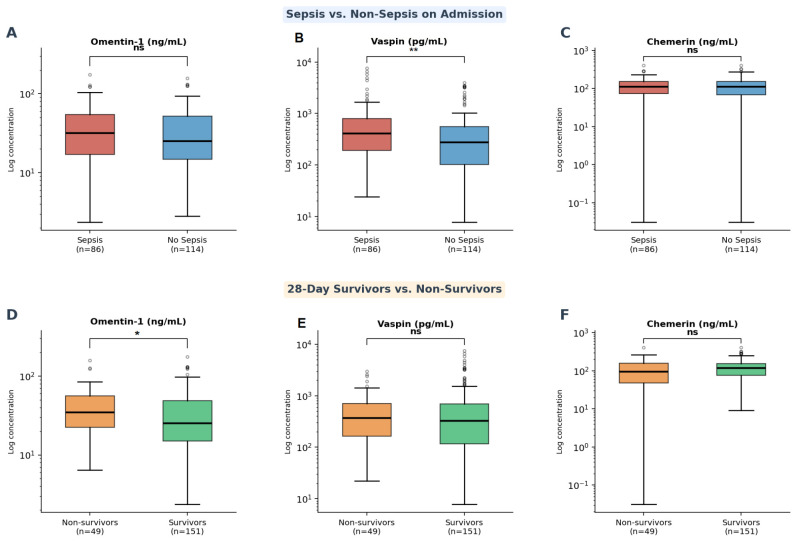
Adipokine concentrations by sepsis status (panels (**A**–**C**)) and 28-day mortality (panels (**D**–**F**)). Boxplots show median, IQR, and 1.5× IQR whiskers. Significance brackets: * *p* < 0.05; ** *p* < 0.01; ns = not significant (Mann–Whitney U). Note: chemerin 28-day mortality comparison (panel (**F**)) is non-significant (*p* = 0.099).

**Figure 2:**
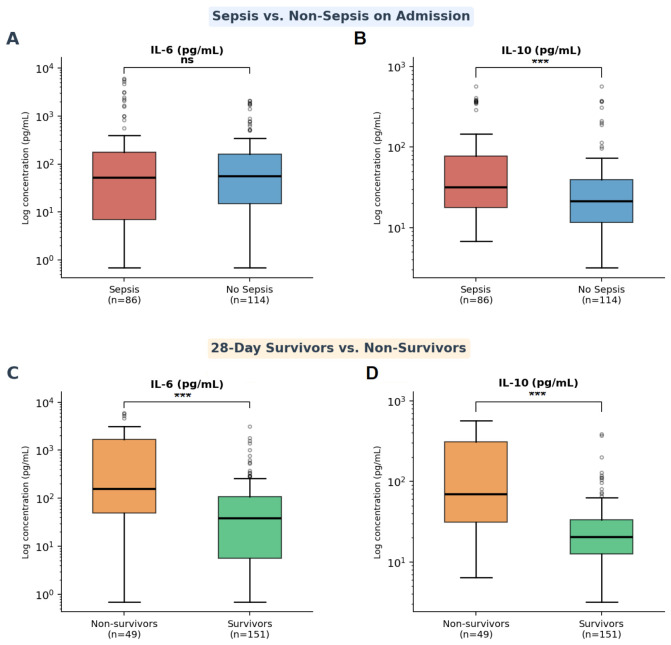
IL-6 and IL-10 concentrations by sepsis status (panels (**A**,**B**)) and 28-day mortality (panels (**C**,**D**)). Reference comparators. Significance brackets: *** *p* < 0.001; ns = not significant (Mann–Whitney U).

Table 2 shows biomarker concentrations by sepsis status (Figure 1 for adipokines; Figure 2 for reference cytokines; biomarker concentrations cross-stratified by sepsis status and 28-day survival are presented in Supplementary Table S1). Among the three adipokines, only vaspin was significantly elevated in septic compared with non-septic patients (406.4 [190.0–799.6] vs. 275.8 [101.8–559.8] pg/mL; *p* = 0.009), representing a 1.5-fold difference. Omentin-1 (31.7 vs. 25.2 ng/mL; *p* = 0.272) and chemerin (110.8 vs. 110.3 ng/mL; *p* = 0.967) did not differ between groups. For reference, IL-10 was significantly higher in septic patients (31.8 vs. 21.3 pg/mL; *p* = 0.001), whilst IL-6 did not differ (51.6 vs. 55.5 pg/mL; *p* = 0.826).

### 3.3. Adipokine Concentrations and 28-Day Mortality

Table 3 presents biomarker concentrations by 28-day mortality, with Figure 1D,F providing visual comparisons for adipokines.

Omentin-1: Non-survivors had significantly higher omentin-1 than survivors (34.4 [22.5–56.1] vs. 25.1 [15.0–48.4] ng/mL; *p* = 0.037). However, this association did not survive adjustment for APACHE II in logistic regression (omentin-1 *p* = 0.295 when co-entered with APACHE II; see Table 4 and Section 3.5). Omentin-1 AUROC for 28-day mortality was 0.599.

Vaspin: Concentrations did not differ between survivors and non-survivors (326.0 vs. 367.8 pg/mL; *p* = 0.640). AUROC was 0.522.

Chemerin: Non-survivors had numerically lower chemerin than survivors (95.4 vs. 117.0 ng/mL), but this difference did not reach statistical significance (*p* = 0.099). Its AUROC was 0.579.

### 3.4. Spearman Correlation Analysis

**Figure 3:**
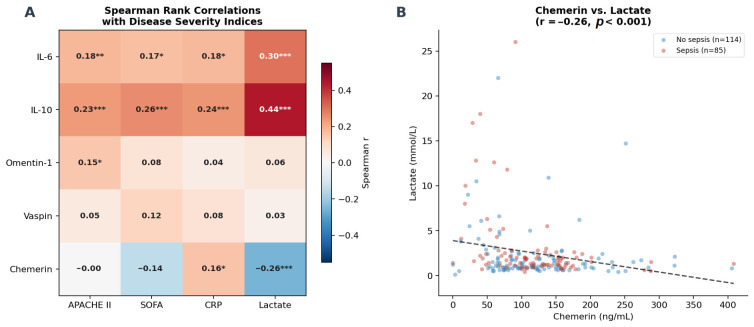
Correlations between biomarkers and disease severity. Panel (**A**): Spearman rank correlation heatmap (* *p* < 0.05; ** *p* < 0.01; *** *p* < 0.001). Panel (**B**): Scatter plot of chemerin vs. lactate, according to sepsis status (full cohort, r = −0.26, *p* < 0.001, with dashed locally estimated scatterplot smoothing (LOWESS) line); within septic patients (red circles) the inverse relationship is r = −0.40, *p* < 0.001.

Figure 3A shows the full Spearman correlation heatmap. The cytokine reference markers showed expected positive associations with all severity indices (IL-10 vs. lactate r = 0.44, *p* < 0.001; IL-6 vs. lactate r = 0.30, *p* < 0.001). Omentin-1 correlated weakly but significantly with APACHE II only (r = 0.15, *p* = 0.036); vaspin showed no significant correlations with any severity index.

Chemerin showed an inverse correlation with lactate across the whole cohort (r = −0.26, *p* < 0.001). Within the septic subgroup alone, this relationship was substantially stronger: chemerin versus SOFA r = −0.43 (*p* < 0.001, n = 86) and versus lactate r = −0.40 (*p* < 0.001, n = 85) (Figure 3B). This inverse relationship is diametrically opposite to the direction observed for IL-6 and IL-10.

### 3.5. Logistic Regression Analysis

Table 4 summarizes the logistic regression analyses. Log-transformed omentin-1 was a significant univariate predictor of 28-day mortality (OR 1.55, 95% CI 1.01–2.40; *p* = 0.047). However, when APACHE II was co-entered, omentin-1 became non-significant (*p* = 0.295) whilst APACHE II remained strongly predictive (*p* < 0.001). This attenuation persisted when SOFA replaced APACHE II (omentin-1 *p* = 0.144; SOFA *p* < 0.001; SOFA AUROC for 28-day mortality 0.745). Further adjustment for corticosteroid use during ICU stay, diabetes, and chronic kidney disease did not materially alter the omentin-1 estimate (*p* = 0.215 in the most adjusted model). For vaspin, the equivalent two-predictor model with APACHE II confirmed non-significance (*p* = 0.940); the same was noted with SOFA in lieu of APACHE. For log-chemerin, the model with APACHE II yielded a significant negative coefficient (*p* = 0.005), consistent with the inverse correlation with severity and higher chemerin in survivors; an analogous result was noted with SOFA in lieu of APACHE, but with *p* = 0.033. Adding log(IL-10) to APACHE II improved model AUC to 0.838.

**Table 4:** Logistic regression analyses for 28-day mortality: univariate and multivariable models adjusted for APACHE II and SOFA. n = 200 patients; 49 events (28-day mortality).

A. Univariate Models					
Predictor	OR [95% CI]	*p* Value	Interpretation		
Log(Omentin-1) alone	1.53 [1.01–2.33]	0.046 *	Significant in isolation; reflects correlation with global inflammatory burden		
Log(Vaspin) alone	1.04 [0.81–1.34]	0.755	Non-significant univariate; vaspin primarily a diagnostic rather than prognostic marker		
Log(Chemerin) alone	0.55 [0.35–0.85]	0.007 **	Significant inverse association; higher chemerin linked to improved survival		
APACHE II alone	1.19 [1.12–1.27]	<0.001 ***	Strong independent predictor; AUROC 0.785—reference standard		
SOFA alone	1.40 [1.23–1.58]	<0.001 ***	Strong independent predictor; AUROC 0.745—reference standard		
Log(IL-10) alone	3.09 [2.13–4.48]	<0.001 ***	Strongest single biomarker; AUROC 0.783, comparable to severity scores		
Log(IL-6) alone	1.58 [1.31–1.90]	<0.001 ***	Significant; AUROC 0.732		
B. Multivariable Models					
Predictor	Combined with APACHE II OR [95% CI] +	*p* Value	Combined with SOFA OR [95% CI]	*p* Value	Interpretation
Log(Omentin-1)	Log(Om): 1.30 [0.80–2.11] APACHE II: 1.18 [1.11–1.26]	0.294 < 0.001 ***	Log(Om): 1.43 [0.89–2.30] SOFA: 1.39 [1.22–1.57]	0.144 < 0.001 ***	Omentin-1 attenuated to non-significance by both severity scores; severity drives outcome. AUC 0.785 (APACHE)/0.751 (SOFA)
Log(Vaspin)	Log(Vp): ns (1.01) APACHE II: 1.19 [1.12–1.27]	0.940 < 0.001 ***	Log(Vp): ns (0.93) SOFA: 1.40 [1.23–1.59]	0.599 < 0.001 ***	Vaspin non-significant in both adjusted models. AUC 0.785 (APACHE)/0.743 (SOFA)—driven entirely by severity score
Log(Chemerin)	Log(Ch): 0.53 [0.34–0.82] APACHE II: 1.20 [1.13–1.28]	0.005 ** < 0.001 ***	Log(Ch): 0.62 [0.40–0.96] SOFA: 1.39 [1.21–1.58]	0.033 * < 0.001 ***	Chemerin retains significant negative association after both APACHE II and SOFA adjustment. AUC 0.814 (APACHE)/0.770 (SOFA)
C. Combined Severity Scores + IL-10 (Best-Performing Models)					
Predictor	Combined with APACHE II OR [95% CI] +	*p* Value	Combined with SOFA OR [95% CI]	*p* Value	Interpretation
Log(IL-10)	Log(IL-10): 2.66 [1.77–3.98] APACHE II: 1.15 [1.08–1.23]	<0.001 *** <0.001 ***	—	—	Best APACHE II-based model; AUROC 0.836. Both IL-10 and APACHE II independently predict 28-day mortality
Log(IL-10) +	—	—	Log(IL-10): 2.66 [1.79–3.95] SOFA: 1.29 [1.13–1.48]	<0.001 *** <0.001 ***	Best SOFA-based model; AUROC 0.821. Both IL-10 and SOFA independently predict 28-day mortality

Log-transformation applied to omentin-1, vaspin, chemerin, IL-10, and IL-6. Chemerin direction: OR < 1 indicates higher chemerin is associated with lower mortality risk; OR, odds ratio; CI, 95% confidence interval; ns, not significant; APACHE II, Acute Physiology and Chronic Health Evaluation II; SOFA, Sequential Organ Failure Assessment; IL, interleukin. * *p* < 0.05; ** *p* < 0.01; *** *p* < 0.001.

### 3.6. ROC Analysis

**Figure 4:**
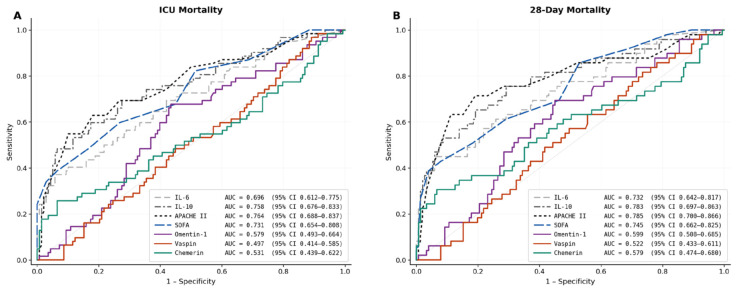
ROC curves for ICU and 28-day mortality. Panel (**A**): ICU mortality. Panel (**B**): 28-day mortality.

Table 5 and Figure 4 present ROC results for both ICU and 28-day mortality. Among adipokines, omentin-1 achieved the highest AUROC (0.579 [95% CI 0.493–0.664] for ICU mortality; 0.599 [95% CI 0.508–0.685] for 28-day mortality). These values fall below the conventional threshold of 0.70 for acceptable clinical discrimination and should be interpreted as reflecting a weak signal at best. Vaspin performed at or near chance, with all 95% CIs crossing 0.50 or approaching it. As established reference markers, IL-10 and IL-6 substantially outperformed all adipokines for 28-day mortality prediction, comparable to APACHE II and SOFA.

### 3.7. Sensitivity Analyses: Corticosteroids, Hepatic Failure, and Diabetes

#### 3.7.1. Corticosteroid Use and Adipokine Concentrations

Corticosteroid use prior to ICU admission (n = 46, 23.0%) did not significantly affect omentin-1, vaspin, or chemerin concentrations at admission (all *p* > 0.22, Mann–Whitney U test; Supplementary Table S2). Corticosteroid use during the ICU stay (n = 104, 52.5%) was more frequent in 28-day non-survivors than survivors (75.5% vs. 44.7%, *p* < 0.001), consistent with administration as escalation therapy in the most critically ill patients rather than prophylactic allocation. Among the three adipokines, only chemerin showed a significant difference by ICU corticosteroid status: patients receiving ICU corticosteroids had lower median chemerin (98.1 [59.1–140.7] ng/mL) compared with those who did not (124.2 [90.2–159.0] ng/mL; *p* = 0.0008). However, in multivariable logistic regression for 28-day mortality including APACHE II, log-chemerin, and ICU corticosteroid use simultaneously, log-chemerin retained its significant negative association with mortality (β = −0.641, *p* = 0.005; OR 0.53 [95% CI 0.34–0.82]), and the corticosteroid term was not independently significant (*p* = 0.066). These results indicate that the lower chemerin in non-survivors is not simply an artefact of corticosteroid use.

Regarding vaspin, its diagnostic signal for sepsis (septic 406.4 pg/mL vs. non-septic 275.8 pg/mL, *p* = 0.009) was preserved in steroid-naive patients (septic 373.4 pg/mL [n = 56] vs. non-septic 257.5 pg/mL [n = 98], *p* = 0.026), although it was attenuated in the steroid-pre-exposed subgroup (septic 475.1 pg/mL [n = 30] vs. non-septic 435.3 pg/mL [n = 16], *p* = 0.526) (Supplementary Table S2). Logistic regression adjusting for pre-admission corticosteroid use confirmed that vaspin (log-transformed) remained a significant predictor of sepsis vs. non-sepsis after adjustment (β = 0.312, *p* = 0.010; OR 1.37 [95% CI 1.08–1.73]), alongside the corticosteroid-pre term (β = 1.189, *p* = 0.001), suggesting that both vaspin elevation and prior corticosteroid exposure contribute independently to the probability of sepsis diagnosis. The attenuation in the pre-exposed subgroup may reflect glucocorticoid-driven vaspin upregulation in non-septic patients receiving steroids before ICU admission, which would reduce the differential between groups.

#### 3.7.2. Hepatic Failure/Cirrhosis

Hepatic failure (defined as clinical cirrhosis or acute hepatic failure; largely the former) was present in four patients (2.1%; three septic, one non-septic). Given the very small number, formal between-group comparisons have limited power, but observed values are presented in Supplementary Table S2 for transparency. Patients with hepatic failure had markedly lower circulating chemerin (47.8 [38.6–55.8] ng/mL) compared to those without (112.8 [73.3–155.9] ng/mL; *p* = 0.009). This is consistent with published data demonstrating reduced chemerin secretion in advanced liver disease due to impaired hepatic and adipocyte synthetic function, and with prior observations that hepatic dysfunction independently suppresses circulating chemerin independently of sepsis severity. Omentin-1 and vaspin were numerically higher in patients with hepatic failure (46.9 ng/mL and 823.8 pg/mL, respectively), but differences did not reach statistical significance (*p* = 0.556 and *p* = 0.104, respectively), likely owing to inadequate power with only four affected patients. Hepatic failure was not independently associated with 28-day mortality in this cohort (*p* = 0.573). These findings are noted as exploratory and should not be over-interpreted; the presence of hepatic failure as a confound for adipokine levels in future ICU studies warrants prospective systematic recording and stratification.

#### 3.7.3. Diabetes and Chronic Kidney Disease

Diabetes was present in 52 patients (26.7%) and was not significantly associated with any of the three adipokine concentrations (all *p* > 0.15) or with 28-day mortality (*p* = 0.642). Chronic kidney disease (CKD; n = 21, 10.8%) was similarly not associated with significant differences in adipokine concentrations at admission (all *p* > 0.10) or with 28-day mortality (*p* = 0.467). Sensitivity logistic models for omentin-1, also including diabetes and CKD as covariates, did not materially alter the omentin-1 estimate (see Section 3.5 and Table 4). These data provide reassurance that the primary adipokine findings are not confounded by these metabolic comorbidities in the present cohort, although the sample size precludes definitive exclusion of interactions.

## 4. Discussion

This study provides a simultaneous evaluation of three understudied adipokines—omentin-1, vaspin, and chemerin—in a prospective, heterogeneous cohort of 200 unselected critically ill patients comprising approximately equal proportions of septic and non-septic disease. The key contribution is not a single dominant biomarker but a characterization of three qualitatively distinct patterns that are biologically informative even where individual predictive performance is modest.

### 4.1. Vaspin: A Diagnostic Marker of Infection, Not Severity

Vaspin was the only adipokine that distinguished septic from non-septic patients on ICU admission (*p* = 0.009), producing a signal that the established cytokine IL-6 failed to provide in the same cohort (*p* = 0.826). This difference likely reflects the broad induction of IL-6 by any major inflammatory stimulus (trauma, post-operative inflammation, burns), whereas vaspin elevation may be more specifically linked to infectious triggers that activate the serine protease cascades responsible for its induction [11]. Vaspin is a marker of the insult—the presence of infection—rather than of severity or prognosis. Indeed, vaspin showed no significant correlation with APACHE II, SOFA, CRP, or lactate, and its AUROC for 28-day mortality was 0.522. This dissociation between a diagnostic and a prognostic signal is clinically meaningful: in the heterogeneous ICU setting where the etiology of systemic inflammation is frequently unclear at admission, a marker that reliably flags infectious etiology has different, complementary utility to a severity marker. Whether vaspin outperforms procalcitonin in this discriminatory role requires head-to-head prospective evaluation.

### 4.2. Omentin-1: A Univariate Mortality Association Driven by Inflammatory Burden

Omentin-1 was significantly elevated in 28-day non-survivors (*p* = 0.037), consistent with the direction reported in focused septic cohorts [9,10]. However, the multivariate logistic regression confirmed that this association is substantially confounded by APACHE II: omentin-1 lost significance entirely (*p* = 0.295) when APACHE II was co-entered. This finding has an important mechanistic interpretation: omentin-1 elevation likely reflects the magnitude of the initial inflammatory stimulus, and APACHE II captures that same severity more directly and completely. The AUROC of 0.599, whilst statistically above chance, represents “poor” discrimination by conventional clinical thresholds (AUC > 0.70 typically required for clinical utility). Future studies incorporating serial measurements [9], where the kinetic change during the first week adds prognostic information beyond baseline, may establish a more robust role. Of note, Gao et al. [15] recently reported a combined midkine/omentin-1 panel with improved prognostic performance in sepsis, highlighting the potential of multi-marker approaches.

### 4.3. Chemerin: A Novel Inverse Relationship with Tissue Hypoperfusion Markers in Sepsis

Chemerin’s most biologically interesting feature in this dataset is its inverse relationship with markers of tissue hypoperfusion within the septic subgroup: Spearman r = −0.43 versus SOFA (*p* < 0.001) and r = −0.40 versus lactate (*p* < 0.001). This pattern was not apparent in the non-septic group and does not translate into a significant mortality signal (28-day *p* = 0.099).

The inverse severity correlation is a genuine and striking finding. It may be interpreted in several ways. First, chemerin activates CMKLR1 on macrophages and plasmacytoid dendritic cells, initiating pro-resolving immune responses [12]; patients with less severe metabolic derangement may have better-preserved immune-regulatory capacity and therefore higher circulating chemerin. Second, extreme lactate elevation (indicating severe hypoperfusion) may coincide with impaired adipose tissue perfusion and thus reduced chemerin secretion—an “exhaustion” hypothesis consistent with the observation that chemerin VAT mRNA expression is decreased in sepsis despite elevated circulating levels [12]. Third, the pathogen composition of a cohort substantially affects chemerin levels: Amend et al. [13] showed that Gram-positive co-infections specifically elevate chemerin. Without pathogen stratification in the present data, this source of heterogeneity cannot be controlled for. Future studies should measure chemerin isoforms separately (active vs. inactive prochemerin), stratify by pathogen, and incorporate serial measurements to disentangle these mechanisms.

### 4.4. The Multi-Biomarker Perspective

The network analyses of Ebihara et al. [16] demonstrated that adipokines act not as standalone predictors but as nodes within a cytokine network. Resistin, IL-6, IL-8, IL-10, and monocyte chemoattractant protein-1 (MCP-1) formed the dominant prognostic cluster in the acute phase of sepsis. The adipokines studied herein (omentin-1, vaspin, chemerin) are likely peripheral to that core network, which is why they individually perform below the threshold of clinical utility. The added value of combining adipokines with cytokines and severity scores in a penalized regression or machine-learning framework—as advocated in recent biomarker reviews [5,17]—has not been tested here and represents the most promising avenue for future work. A specific confounder that warrants explicit discussion is corticosteroid use during ICU stay, which was significantly more frequent in 28-day non-survivors (75.5% vs. 44.7%, *p* < 0.001). Corticosteroids are known modulators of adipokine secretion: they acutely elevate vaspin and suppress adiponectin, and may alter omentin-1 expression through PPAR-γ pathways. Because corticosteroids in this cohort were administered as rescue therapy in the most critically ill patients (i.e., driven by severity), they represent an intermediary on the causal pathway between severity and outcome rather than an independent confounder in the strict sense. Nevertheless, sensitivity models adjusting for corticosteroid use did not materially change any adipokine coefficient, and results were consistent, providing some reassurance that corticosteroid exposure does not account for the observed associations. Similarly, the higher Charlson Comorbidity Index in septic patients (median 4 vs. 3, *p* < 0.01) and the enrichment of COPD in the septic subgroup (25.6% vs. 4.5%, *p* < 0.001)—which are expected given that pulmonary comorbidities predispose to respiratory sepsis—were documented and are presented in Table 1. No comorbidity was individually associated with 28-day mortality after severity adjustment.

### 4.5. Strengths and Limitations

Strengths of this work include: (i) simultaneous measurement of all five biomarkers in the same admission blood draw, eliminating pre-analytical variability between samples; (ii) inclusion of both septic and non-septic critically ill patients, representing the most clinically realistic ICU population and enabling internal comparison without a healthy-volunteer control group that would be physiologically non-equivalent; (iii) both APACHE II and SOFA as internal reference standards, anchoring biomarker AUROCs to validated clinical benchmarks; (iv) prospective data collection with complete 28-day mortality follow-up for all 200 patients (zero loss to follow-up); and (v) all ELISAs performed in a single batch, in duplicate, by one blinded operator, minimizing run-to-run and operator variability.

Limitations must be acknowledged. First, this is a single-center study with a cross-sectional biomarker design (single admission time-point only); serial measurements, which are essential to characterize kinetics and track counter-regulatory trajectories, were not available. Second, the analytic sample of 49 28-day deaths limits the stability of multivariate models; per the widely cited guideline of 10 events per predictor, a maximum of four or five predictors can be reliably estimated, which prevents construction of comprehensive confounder-adjusted models with many covariates simultaneously. Third, BMI data were not systematically available for all patients; because adipokine concentrations are influenced by body composition, residual confounding by adiposity cannot be excluded. We acknowledge this limitation. Fourth, the ELISA platforms measure total circulating chemerin without isoform differentiation; given that bioactive chemerin isoforms differ functionally from prochemerin, this represents a relevant measurement limitation. Fifth, structured pathogen-level data (organism, Gram-status, culture site) were not available for systematic stratification in the septic subgroup; this precludes analysis of pathogen-specific adipokine responses. Sixth, corticosteroid use during ICU stay was high in non-survivors (75.5%) versus survivors (44.7%; *p* < 0.001), reflecting their receipt as rescue therapy in the sickest patients rather than a priori allocation; although we have added corticosteroid use as a sensitivity covariate in the revised multivariable analyses, residual confounding cannot be ruled out in this observational design. Seventh, the single-center design and the specific mixed medical-surgical ICU case-mix may limit generalizability to other ICU populations.

## 5. Conclusions

In 200 unselected critically ill ICU patients, three adipokines exhibited qualitatively distinct patterns that are biologically informative. Vaspin elevation at ICU admission provides a diagnostic signal for the presence of sepsis—information that IL-6 does not provide in the same cohort—but carries no prognostic value. Omentin-1 is univariately associated with 28-day mortality but loses significance after APACHE II adjustment, indicating its signal is driven by baseline physiological severity rather than independent pathophysiology. Chemerin’s inverse correlation with lactate and SOFA within septic patients is the most novel finding, suggesting a pro-resolving or counter-regulatory role in established infection that merits dedicated mechanistic investigation. Individually, these adipokines add limited predictive value above APACHE II or IL-10. Their biological orthogonality to classical cytokines, however, makes them candidates for multi-biomarker panel development in future prospective multicenter studies.

## Figures and Tables

**Table 1:** Baseline characteristics of 200 critically ill ICU patients stratified by sepsis status on admission.

Variable	All (n = 200)	Sepsis on Admission (n = 86) #	No Sepsis on Admission (n = 114)
Age, years—median [IQR]	63 [52–71]	66 [57–73]	60 [49–68]
Male sex—n (%)	116 (58.0)	52 (60.5)	64 (56.1)
APACHE II—median [IQR]	15 [11–20]	17 [14–22] *	13 [9–17]
SOFA—median [IQR]	8 [5–10]	8 [6–10] *	7 [5–9]
Lactate, mmol/L—median [IQR]	1.4 [0.9–2.3]	1.7 [1.1–2.5] *	1.1 [0.8–2.1]
CRP, mg/dL—median [IQR]	9.8 [3.0–18.8]	15.4 [6.4–26.1] ***	5.8 [1.6–12.6]
ICU mortality—n (%)	62 (31.0)	38 (44.1) ***	24 (21.0)
28-day mortality—n (%)	49 (24.5)	29 (33.7) *	20 (17.5)
Diabetes mellitus—n (%)	52 (26.0)	24 (27.9)	28 (24.6)
Chronic kidney disease—n (%)	21 (10.5)	12 (13.9)	9 (7.8)
COPD—n (%)	26 (13.0)	21 (24.4) ***	5 (4.4)
Hypertension—n (%)	84 (42.0)	37 (43.0)	47 (41.2)
CAD—n (%)	29 (14.5)	15 (17.4)	14 (12.2)
Cancer—n (%)	46 (23.0)	16 (18.6)	30 (26.3)
Hepatic failure/cirrhosis—n (%)	4 (2.1)	3 (3.6)	1 (0.9)
Charlson Comorbidity Index—median [IQR]	3 [2–6]	4 [3–6] **	3 [1–5]
Corticosteroids prior to ICU—n (%)	46 (23.0)	30 (34.9) **	16 (14.0)
Corticosteroids during ICU—n (%)	104 (52.5)	59 (69.4) ***	45 (39.5)

IQR, interquartile range; APACHE II, Acute Physiology and Chronic Health Evaluation II; SOFA, Sequential Organ Failure Assessment; CRP, C-reactive protein; COPD, chronic obstructive pulmonary disease; CAD, coronary artery disease. *p* values from Mann–Whitney U test (continuous) or chi-squared (categorical). * *p* < 0.05; ** *p* < 0.01; *** *p* < 0.001 vs. no-sepsis group. Between-group differences that were not significant (*p* > 0.05): diabetes (*p* = 0.71), chronic kidney disease (*p* = 0.25), hepatic failure/cirrhosis (*p* = 0.43), CAD (*p* = 0.31), cancer (*p* = 0.33), hypertension (*p* = 0.98). Hepatic failure/cirrhosis was present in only 4 patients; between-group comparison has very limited power. BMI data were not systematically available and are not reported. # Infection site: lung, N = 46; abdomen, N = 14; blood, N = 13; CNS = 7; soft tissue, N = 4; urinary, N = 2.

**Table 2:** Admission biomarker concentrations by sepsis status on admission.

Biomarker	Sepsis on Admission (n = 86) Median [IQR]	No Sepsis on Admission (n = 114) Median [IQR]	*p* Value
Adipokines (primary study markers)			
Omentin-1 (ng/mL)	31.7 [17.1–54.3]	25.2 [14.8–51.7]	0.272
Vaspin (pg/mL)	406.4 [190.0–799.6]	275.8 [101.8–559.8]	0.009
Chemerin (ng/mL)	110.8 [73.8–154.6]	110.3 [68.0–154.2]	0.967
Cytokines (reference comparators)			
IL-6 (pg/mL)	51.6 [7.0–176.7]	55.5 [14.9–161.9]	0.826
IL-10 (pg/mL)	31.8 [17.8–77.9]	21.3 [11.6–39.2]	0.001

Values are median [IQR]. Two-sided Mann–Whitney U test.

**Table 3:** Biomarker concentrations by 28-day mortality.

Biomarker	28-Day Survivors (n = 151) Median [IQR]	28-Day Non-Survivors (n = 49) Median [IQR]	*p* Value
Adipokines			
Omentin-1 (ng/mL)	25.1 [15.0–48.4]	34.4 [22.5–56.1]	0.037
Vaspin (pg/mL)	326.0 [115.3–686.4]	367.8 [163.1–698.9]	0.640
Chemerin (ng/mL)	117.0 [75.1–154.1]	95.4 [48.3–157.6]	0.099 (trend only)
Cytokines			
IL-6 (pg/mL)	38.2 [5.7–109.0]	156.0 [49.2–1686.0]	<0.001
IL-10 (pg/mL)	20.3 [12.6–33.5]	69.5 [31.3–309.8]	<0.001

Values are median [IQR]. Two-sided Mann–Whitney U test.

**Table 5:** ROC analysis for ICU and 28-day mortality (AUC, sensitivity, specificity at Youden index threshold).

Biomarker	AUC (ICU)	Sens (ICU)	Spec (ICU)	AUC (28-d)	Sens (28-d)	Spec (28-d)
Adipokines						
Omentin-1	0.579 [0.493–0.664]	0.68	0.579 [0.493–0.664]	0.599 [0.508–0.685]	0.69	0.55
Vaspin	0.503 [0.415–0.590]	1.00	0.09	0.522 [0.433–0.611]	0.82	0.26
Chemerin	0.531 [0.439–0.622]	0.26	0.93	0.579 [0.461–0.692]	0.31	0.93
Reference Markers						
IL-6	0.696 [0.612–0.775]	0.37	0.94	0.732 [0.642–0.817]	0.45	0.94
IL-10	0.758 [0.676–0.833]	0.60	0.83	0.783 [0.697–0.863]	0.76	0.71
APACHE II	0.764 [0.688–0.837]	0.63	0.82	0.785 [0.700–0.866]	0.71	0.81
SOFA	0.719 [0.654–0.808]	0.58	0.79	0.745 [0.662–0.825]	0.67	0.79

AUC, area under the receiver-operating characteristic curve (point estimate [95% bootstrap confidence interval, 2000 iterations]); Sens, sensitivity; Spec, specificity at optimal Youden index threshold. APACHE II, SOFA, IL-6, and IL-10 are reference comparators. AUROCs < 0.70 indicate poor standalone discrimination. See also Supplementary Table S1 for biomarker values cross-stratified by sepsis status and 28-day survival.

## Data Availability

The data presented in this study are available on request from the corresponding author. The data are not publicly available due to patient privacy restrictions.

## Supplementary Materials

The following supporting information can be downloaded at: https://www.mdpi.com/article/10.3390/biomedicines14061324/s1. Table S1. Biomarker Concentrations Stratified by Sepsis Status and 28-Day Survival; Table S2. Sensitivity Analyses: Adipokine Concentrations by Corticosteroid Use and Hepatic Failure Status.
