# Supplementary material for: Circulating Novel Adipokines in Critically Ill Patients with and Without Sepsis
Source: Biomedicines. 2026 Jun 11;14(6):1324. doi: 10.3390/biomedicines14061324 (PMC13297440; doi:10.3390/biomedicines14061324)
Supplement: Supplementary file 1 [file biomedicines-14-01324-s001.zip › supplementary table S1 june 01 2026.pdf]

Supplementary Material

Supplementary Table S1. Biomarker Concentrations Stratified by Sepsis Status and 28-Day Survival

Values are median [interquartile range]. p values from two-sided Mann–Whitney U test. S-NS, septic non-survivors; S-S, septic survivors; NS-NS, non-septic non-survivors; NS-S, non-septic survivors. n (S-NS) = 29; n (S-S) = 57; n (NS-NS) = 20; n (NS-S) = 94.

Biomarker	S-NS (n=29)	S-S (n=57)	NS-NS (n=20)	NS-S (n=94)
Omentin-1 (ng/mL)	34.1 [22.6–56.1]	30.3 [16.7–40.6] *	37.1 [20.9–55.6]	24.4 [14.6–50.3]
p (survivors vs. non-survivors within stratum)	*Septic: p = 0.277*		*Non-septic: p = 0.111*	
Vaspin (pg/mL)	475.6 [267.6–730.4]	371.8 [168.2–938.4]	214.5 [90.8–480.5]	304.6 [105.2–570.5]
p (survivors vs. non-survivors within stratum)	*Septic: p = 0.421*		*Non-septic: p = 0.392*	
Chemerin (ng/mL)	91.1 [39.9–124.2]	129.6 [83.4–158.0] ***	119.6 [62.6–219.8]	110.3 [69.3–149.8]
p (survivors vs. non-survivors within stratum)	*Septic: p = 0.004*		*Non-septic: p = 0.539*	
IL-6 (pg/mL)	117.8 [53.7–2091.3]	22.8 [5.1–90.7] ***	340.2 [43.5–1091.7]	49.6 [10.3–122.6] ***
p (survivors vs. non-survivors within stratum)	*Septic: p < 0.001*		*Non-septic: p = 0.001*	
IL-10 (pg/mL)	85.4 [32.6–357.7]	21.2 [16.4–53.1] ***	56.9 [25.0–194.8]	19.6 [10.9–32.2] ***
p (survivors vs. non-survivors within stratum)	*Septic: p < 0.001*		*Non-septic: p < 0.001*	

*S-NS, septic non-survivors; S-S, septic survivors; NS-NS, non-septic non-survivors; NS-S, non-septic survivors. * p < 0.05; *** p < 0.001 vs. non-survivors within the same sepsis stratum (Mann–Whitney U test). The chemerin signal (septic non-survivors vs. survivors, p = 0.004) supports the hypothesis of a counter-regulatory role in established infection.*
