# Supplementary material for: Circulating Novel Adipokines in Critically Ill Patients with and Without Sepsis
Source: Biomedicines. 2026 Jun 11;14(6):1324. doi: 10.3390/biomedicines14061324 (PMC13297440; doi:10.3390/biomedicines14061324)
Supplement: Supplementary file 1 [file biomedicines-14-01324-s001.zip › Supplementary_Table_S2 june 01 2026.pdf]

Supplementary Table S2. Sensitivity Analyses: Adipokine Concentrations by Corticosteroid Use and Hepatic Failure Status

All values are median [IQR]. p values from two-sided Mann–Whitney U test. Statistically significant p values ($p < 0.05$) are shown in bold.

Table S2A. Biomarker concentrations by corticosteroid use (prior to ICU admission and during ICU stay)

Biomarker	No prior steroids (n = 154)	Prior steroids (n = 46)	p value	No ICU steroids (n = 96)	ICU steroids (n = 104)	p value
Omentin-1 (ng/mL)	29.5 [15.9–55.6]	29.6 [15.7–39.7]	0.541	29.2 [17.7–50.0]	29.7 [14.8–55.5]	0.568
Vaspin (pg/mL)	317.1 [115.1–694.5]	461.2 [210.9–678.8]	0.227	308.2 [114.2–616.1]	358.8 [155.4–701.8]	0.368
Chemerin (ng/mL)	110.4 [73.3–154.2]	112.2 [60.3–154.3]	0.564	124.2 [90.2–159.0]	98.1 [59.1–140.7]	0.001
IL-6 (pg/mL)	65.4 [12.1–225.3]	34.8 [10.8–122.6]	0.218	41.8 [9.6–143.2]	71.2 [13.5–253.6]	0.113
IL-10 (pg/mL)	23.4 [14.6–54.3]	22.1 [15.4–49.9]	0.812	20.2 [13.8–40.1]	28.8 [17.2–78.4]	0.055

** Only chemerin showed a significant difference by ICU corticosteroid status ($p = 0.001$). No adipokine differed significantly by pre-admission corticosteroid use. See Section 3.7.1 for multivariable adjustment.*

Table S2B. Biomarker concentrations in patients with vs. without hepatic failure/cirrhosis

Biomarker	No hepatic failure (n = 190)	Hepatic failure/cirrhosis (n = 4)	p value *
Omentin-1 (ng/mL)	29.5 [15.7–53.7]	46.9 [24.7–74.1]	0.556
Vaspin (pg/mL)	329.1 [123.8–661.0]	823.8 [618.1–1020.9]	0.104
Chemerin (ng/mL)	112.8 [73.3–155.9]	47.8 [38.6–55.8]	0.009
IL-6 (pg/mL)	55.5 [10.9–180.1]	26.4 [18.6–47.2]	0.351
IL-10 (pg/mL)	24.3 [15.0–57.3]	22.5 [19.0–28.4]	0.749

** Mann–Whitney U test. n = 4 patients with hepatic failure; statistical comparisons have very limited power and results should be considered exploratory. Chemerin was significantly lower in hepatic failure patients ($p = 0.009$), consistent with published literature on reduced adipokine synthesis in advanced liver disease.*

Table S2C. Vaspin (pg/mL) diagnostic signal for sepsis vs. non-sepsis, stratified by prior corticosteroid use

Corticosteroid strata	Septic patients median [IQR]	n	Non-septic patients median [IQR]	n	p value
Steroid-naïve (no prior steroids)	373.4 [172.2–833.1]	56	257.5 [101.1–543.9]	98	0.026
Steroid-exposed (priorsteroids)	475.1 [261.6–780.2]	30	435.3 [212.7–658.4]	16	0.526

The sepsis vs. non-sepsis difference in vaspin is preserved in steroid-naïve patients ($p = 0.026$) but attenuated in those with prior steroid exposure ($p = 0.526$), consistent with glucocorticoid-driven vaspin upregulation in non-septic patients receiving pre-ICU corticosteroids. After logistic regression adjusting for prior corticosteroid use, log-vaspin remained a significant predictor of sepsis ($\beta = 0.312$, $p = 0.010$; OR 1.37 [95% CI 1.08–1.73]).
